# Dazed and Confused: NK Cells

**DOI:** 10.3389/fimmu.2019.02235

**Published:** 2019-09-20

**Authors:** Timothy E. O'Sullivan

**Affiliations:** Department of Microbiology, Immunology, and Molecular Genetics, David Geffen School of Medicine at University of California, Los Angeles, Los Angeles, CA, United States

**Keywords:** NK cells, group 1 ILCs, development, heterogeneity, ILC1

## Introduction

Innate lymphoid cells (ILCs) are rapid producers of both proinflammatory and regulatory cytokines in response to local injury, inflammation, pathogen infection, or commensal microbiota perturbation (1). Because most ILCs have been shown to be tissue-resident during homeostasis (with the exception of circulating NK cells) in almost all organs analyzed, their ability to quickly respond to tissue stress and inflammation underpins their critical role in regulating tissue homeostasis and repair during infection or injury (2–4). Recent evidence has suggested that mature ILCs can be further classified into group 1, 2, and 3 ILCs based on different expression of transcription factors, cell surface markers, and effector cytokines (1). Mouse group 1 ILCs, which include natural killer (NK) cells and ILC1, were initially distinguished from other ILCs based on their constitutive expression of the transcription factor *Tbx21* (T-bet), co-expression of activating receptors NKp46 and NK1.1, and production of interferon (IFN)-γ following activation (5). In humans, group 1 ILCs are harder to definitively differentiate from other ILCs due to the lack of lineage defining markers and reported functional plasticity amongst group 2 and group 3 ILCs (6).

ILC1 are recently discovered tissue-resident sentinels that function to protect the host from bacterial and viral pathogens at initial sites of infection (2, 7, 8). ILC1 rapidly produce IFN-γ following local dendritic cell activation and interleukin (IL)-12 production to limit viral replication and promote host survival before the recruitment of circulating lymphocytes into infected tissue (2). Unlike ILC1, NK cells can be recruited from the circulation into the parenchyma of infected or cancerous tissues where they display potent perforin-dependent cytotoxicity in addition to rapid IFN-γ production (9, 10). However, persistent inflammatory signals can also lead to unrestrained activation of group 1 ILCs during obesity and inflammatory bowel disease (IBD) (3, 11–14). While these studies suggest important roles for group 1 ILCs during host protection and pathology, gaps in evidence have inhibited the ability of recent studies to definitively distinguish between the roles of ILC1 and NK cells in these contexts.

## Group 1 ILC Phenotypic and Functional Heterogeneity

NK cells, the founding member of ILCs, were initially defined based on the cell surface expression of NK1.1 in mouse or CD56 in human with the absence of cell surface expression of other lineage (Lin) defining markers including CD3, CD14, CD19, and TCR proteins (15). In subsequent mouse studies over the last 30 years, Lin^−^NK1.1^+^ cells were found to be heterogeneous for the expression of activating and inhibitory Ly49 receptors, cell surface integrins [α1β1 (CD49a), α2β1 (CD49b), αEβ7 (CD103)], cell surface proteins (TRAIL, CD69, CD27, CD11b), transcription factors (Eomes), chemokine receptors (CXCR6), and cytokine receptors (IL-7Rα) in various organs (1, 16). Similarly, human Lin^−^CD56^+^ cells have been reported to be heterogeneous for the expression of transcriptions factors (EOMES and T-BET), cell surface markers (CD49a, CD56, CD16, NKp80, CXCR6, IL-7Rα, CD94, CD69, NKp44), and cytotoxic molecules (Perforin) (1, 16). Early studies concluded that cells with an alternative cell surface or transcription factor phenotype from putative mature NK cells (mouse: Lin^−^NK1.1^+^T-bet^+^Eomes^+^CD49b^+^; human: Lin^−^ IL-7Rα^−^CD56^dim^CD16^+^) in peripheral organs and blood likely represented immature NK (iNK) cells (17–21). This hypothesis is supported by studies demonstrating that subsets of developing mouse NK cells can be distinguished based on CD27 and CD11b expression (22, 23). Similarly, previous studies have suggested that CD56^bright^CD16^−^ human NK cells in the blood may be immature precursors to CD56^dim^CD16^+^ mature NK cells (18, 19). However, whether other phenotypic differences observed in mouse and human group 1 ILCs are due to tissue-specific microenvironments, distinct lineages of cells, or developmental/activation states of NK cells is still under considerable debate and investigation.

Insight into these questions came shortly after the identification of Lin^−^IL-7Rα^+^ “helper” ILCs. Specifically, genetic evidence suggested that *Tbx21*-dependent IL-7Rα^+^Tbet^+^Eomes^−^NK1.1^+^NKp46^+^ “ILC1” in the small intestine did not require Eomes for their development, whereas NK cells did require Eomes (7). A recent study further supported these initial data by using *Eomes*-GFP reporter mice to generate core transcriptional signatures of Eomes^−^ ILC1 and Eomes^+^ NK cells from 4 independent tissues. The identified core ILC1 signature led to the discovery of the inhibitory receptor CD200r1 as a stable marker expressed by ILC1 but not NK cells during homeostasis and inflammation (2). Additional lineage tracing experiments suggested that CD200r1^+^Eomes^−^CD49b^−^ group 1 ILCs constituted a stable lineage during homeostasis, distinct from CD200r1-Eomes^+^CD49b^+^ mature NK (mNK) cells (2, 7, 24). Functional evidence suggestive of distinct group 1 ILCs in peripheral organs was supported by the findings that T-bet^+^Eomes^−^CD49b^−^ group 1 ILCs (in addition to ILC2 and ILC3) were long-term tissue-resident cells, whereas Eomes^+^CD49b^+^ mNK cells were derived from the circulation in almost all organs tested in mouse parabiosis experiments (2, 4). Similarly, in one human study a subset of donor liver CXCR6^+^ group 1 ILCs was found to be maintained up to 13 years post-liver transplant while donor CXCR6^−^ NK cells were absent, suggesting that a subset of long-term tissue-resident CXCR6^+^ group 1 ILCs are conserved in mammals (25). Furthermore, CD49b^−^Eomes^−^ group 1 ILCs with a phenotype consistent with ILC1 in the liver express higher levels of TRAIL than mNK cells at steady state, and these ILC1 can produce higher levels of tumor necrosis factor (TNF)-α and IFN-γ following activation *ex vivo* (2, 17, 20, 24). While ILC1 in the small intestine were observed to have poor cytotoxicity and liver group 1 ILCs with a phenotype consistent with ILC1 express lower levels of granzymes A/B and perforin at steady state compared to NK cells (7, 24), peripheral ILC1 express higher transcript levels of granzyme C in addition to TRAIL and may kill target cells through alternative mechanisms (2, 24, 26–28). However, it will be important for future studies to determine whether perforin-independent killing mechanisms can be used as definitive criteria to functionally separate ILC1 from NK cells across all mouse and human tissues. Thus, significant phenotypic and functional heterogeneity has been demonstrated in group 1 ILCs; however, it is still unclear to what extent these individual pieces of evidence can be used in isolation to define group 1 ILC subsets.

## Developmental and Activation States of Group 1 ILCs

Collective reports have demonstrated that iNK cells in mouse bone marrow and periphery can express Ly49 receptors, CD49a, CD90, TRAIL, CD69, and Eomes, and lack CD49b expression (3, 21, 29–31). Upon adoptive transfer into lymphopenic mice, iNK cells can induce CD49b expression and retain Eomes expression (3). During activation, mNK cells can induce expression of CD49a, CD69, TRAIL, and CD90 while also decreasing Eomes expression (2, 17, 29, 32), suggesting that iNK and mNK cell phenotypes can overlap with other reported group 1 ILC phenotypes based on these markers. Consistent with these findings, NK cells can repress Eomes expression and induce CD49a, TRAIL, and CD103 in response to TGFβ and IL-2 stimulation *ex vivo* (33, 34). These key findings make the current dogma of utilizing CD49a, CD49b, and Eomes expression in Lin^−^T-bet^+^NK1.1^+^NKp46^+^ cells insufficient to distinguish between group 1 ILC subsets and activation or developmental states of NK cells. Furthermore, adipose and small intestine iNK cells have also been found to be short-term (1 month), but not long term (4 months) tissue-resident in mouse parabiosis experiments (3), suggesting that short-term parabiosis (2 weeks-1 month) experiments are not sufficient to distinguish iNK cells from ILC1 without additional evidence. Thus, there is currently insufficient evidence to conclude that T-bet^+^ group 1 ILCs with the phenotype of CD49a^+^CD49b^+^Eomes^+^NK1.1^+^ are either tissue-resident NK (trNK) cells or transitional states of group 1 ILCs, because these cells may be activated NK cells in the tissue parenchyma following recruitment from circulation. Furthermore, CD49a^+^CD49b^−^Eomes^+^NK1.1^+^ cells may not represent a transitional subset of group 1 ILC, but instead may represent iNK cells in peripheral tissues, although further lineage tracing experiments will be necessary to clarify these issues in the field.

In the healthy state, mature human group 1 ILCs have been described to be heterogeneous for cell surface expression of CD56, CD16, and NKp80 in peripheral tissues (35). However, CD56 can be expressed on ILC progenitor populations and ILC3 in the tonsil (36), and may be downregulated during activation in a similar manner to CD16 and NKp80 (37–39). Thus, to date there are no known stable cell surface markers that can unequivocally distinguish between human mNK cells (or their developmental intermediates, which may be tissue-resident) and other proposed group 1 ILCs in inflamed human tissues, because activated mNK cells can lose expression of these cell surface markers during inflammation.

### Mouse Group 1 ILC Development

Recent unbiased chromatin accessibility studies in mice suggest that NK cells can be defined epigenetically as a distinct ILC lineage through the enrichment of accessible T-bet and Eomes binding sites compared to other leukocytes (40). Similarly, mNK and iNK cells require Eomes for their development (2, 20, 41), suggesting that Eomes may be the master transcription factor that defines NK cell lineage identity in mice during homeostasis. In support of this hypothesis, mNK cells in the peritoneum, liver, spleen, salivary gland, and adipose tissue were all found to have a cell-intrinsic developmental requirement for Eomes and T-bet (2), arguing against tissue-specific transcription factor developmental requirements for mNK cells. While certain studies have observed that mNK cell numbers are normal in the absence of T-bet (7, 8, 42), it has been demonstrated previously that *Tbx21*^−/−^ NK cells display an immature phenotype and are functionally deficient (3, 43–45). Therefore, because *Tbx21* is required for optimal mature ILC1 and mNK development (2, 3, 46), *Rag2*^−/−^ × *Tbx21*^−/−^ mice are not a suitable model to test for the contributions of mature group 1 ILCs *in vivo*.

The transcription factors *Id2* and *Nfil3* have also been shown to be required for mature mouse ILC1 and NK cell development (47, 48). Certain studies have identified “tissue-resident NK cells,” “salivary gland ILCs,” and “type 1 ILCs” based on their development in the absence of *Nfil3* (27, 33, 49). However, similar subsets have been also found to be *Nfil3*-dependent in a cell-intrinsic manner in other studies (2, 50). Because mNK cells can develop in an *Nfil3*-independent manner during virus-induced inflammation and aging (33, 51), analysis of *Nfil3*^−/−^ mice is likely not sufficient to define group 1 ILC subsets due to these caveats. Previous studies have also utilized Zbtb16 fate-mapping studies and Id2 reporter mice to identify a common helper ILC precursor population that gives rise to all tissue-resident ILCs, but not mNK cells, to argue that ILC1 comprise a developmental lineage distinct from NK cells (7, 52, 53). However, a recent study using dual *Zbtb16* and *Id2* reporter mice demonstrated that both NK cells and ILC1 can develop from a *Id2*^+^*Zbtb16*^+^ shared precursor, suggesting that these transcription factors alone cannot be used to identify different group 1 ILC subsets during ontogeny (54). Instead, several studies have identified the transcription factor *Zfp683* (Hobit) as highly expressed in peripheral ILC1 compared to mNK cells (2, 55, 56). *Zfp683*^−/^^−^ mice display a loss of liver ILC1 but not other ILC populations (including ILC1 in other tissues) (2, 55), suggesting that mature liver ILC1 have a unique developmental pathway from other mouse ILCs. While developmental dependence on Eomes expression can be used to identify NK lineage cells in peripheral organs of mice, there is still no definitive evidence that a single transcription factor can define the development of other group 1 ILC subsets across all mouse tissues.

## Discussion

While collective evidence supports the hypothesis that mouse group 1 ILCs are composed of *Eomes*-dependent iNK and mNK cells, their activation or developmental states may be mistaken for novel subsets of group 1 ILCs. *Eomes*-independent ILC1 have been shown through single- cell sequencing, parabiosis, lineage tracing, and transcription factor deficient mouse experiments to be a distinct lineage of group 1 ILCs, and not a developmental or activation state of NK cells. In human tissues, there is currently no definitive evidence that can distinguish between developmental or activation states of group 1 ILCs during inflammation. Single cell sequencing studies will be needed to determine the extent of group 1 ILC heterogeneity in various peripheral tissues, and to identify stable markers that can distinguish between stable subsets of group 1 ILCs through lineage tracing in humanized mouse models.

## Author Contributions

The author confirms being the sole contributor of this work and has approved it for publication.

### Conflict of Interest Statement

The author declares that the research was conducted in the absence of any commercial or financial relationships that could be construed as a potential conflict of interest.
